# Ketamine-Induced Uropathy in a High-Prevalence Region: Knowledge, Diagnostic Practices, and Treatment Patterns Among Primary and Secondary Care Providers

**DOI:** 10.7759/cureus.86373

**Published:** 2025-06-19

**Authors:** Zakaria W Shkoukani, Praveen Gopi, Lauren Tate, Alina Calmuc, Ahmad Omar, Mohamed I Abdulmajed

**Affiliations:** 1 Department of Urology, Mersey and West Lancashire Teaching Hospitals NHS Trust, Prescot, GBR

**Keywords:** geographic variations in drug misuse, ketamine-induced uropathy, multidisciplinary management, primary care awareness, secondary care practices

## Abstract

Ketamine-induced uropathy (KIU) is an emerging consequence of recreational ketamine misuse, often leading to progressive urinary tract dysfunction. Despite increasing prevalence, clinician awareness and standardized management remain limited. This study evaluated knowledge and practices among healthcare professionals in Cheshire and Merseyside, a region in the United Kingdom with high KIU incidence.

A convergent mixed-methods observational study was conducted, comprising a cross-sectional knowledge, attitudes, and practice (KAP) survey of 107 primary and secondary care professionals, as well as a retrospective review of 65 KIU patients over six months at a regional high-volume urology center. Survey responses underwent descriptive and comparative statistical analysis; clinical data were reviewed for diagnostic patterns, interventions, and outcomes.

Secondary care professionals demonstrated significantly greater awareness of KIU than primary care counterparts (p=0.005), though overall familiarity with British Association of Urological Surgeons (BAUS) guidelines was limited. Primary care respondents expressed a lack of confidence in managing KIU and were more likely to defer care to secondary services. A notable proportion believed that law enforcement should be involved in management, reflecting broader public health concerns. High rates of non-attendance (41.5%) highlighted the need for early psychosocial intervention. General practitioners (GPs) expressed strong interest in targeted education to improve community-based care.

Awareness and management of KIU remain inconsistent across healthcare settings. The recent BAUS consensus guidelines offer a unified framework that, if widely adopted, could standardize care and curb disease progression. Future directions include establishing multidisciplinary clinics and exploring urological subspecialization in KIU. Enhanced primary care education and earlier intervention are essential to improving patient outcomes and reducing long-term morbidity.

## Introduction

Ketamine, a phencyclidine derivative, is a short-acting non-competitive inhibitor of the N-methyl-D-aspartate (NMDA) receptor, widely utilized in clinical practice for its anesthetic and analgesic properties, particularly in the management of neuropathic and malignancy-associated pain [1,2]. However, its psychotropic and dissociative effects have led to widespread misuse as a recreational drug, particularly within nightlife and club cultures across the United Kingdom, Europe, and Asia [3].

The primary metabolite of ketamine, norketamine, is formed in the liver and excreted renally. This metabolite has been implicated in significant detrimental effects on both the hepatobiliary and urinary systems [4]. Chronic ketamine misuse can result in persistent inflammation of the urothelium, leading to a clinical syndrome known as ketamine-induced uropathy (KIU). This condition encompasses a range of upper and lower urinary tract symptoms (LUTS), including urgency, frequency, painful hematuria, urge incontinence, and pelvic or flank pain [5]. Without timely cessation of ketamine use, affected individuals are at risk of progressive renal impairment, culminating in renal failure [6].

Since the initial description of KIU in 2007, the incidence of reported cases has risen markedly. In the United Kingdom, the prevalence of ketamine misuse and related uropathies among individuals aged 16 to 24 years doubled between 2010 and 2020 [7]. This trend presents a growing diagnostic and therapeutic challenge for clinicians across both primary and secondary care settings. Emerging case reports have also linked chronic ketamine use with severe complications such as bladder malignancy and even death [7,8]. Alarmingly, some young patients require reconstructive urological surgery, highlighting the urgency of addressing this evolving public health issue.

This study aims to evaluate the current level of knowledge and awareness regarding KIU among primary and secondary healthcare professionals in a high-prevalence region of the United Kingdom. It also seeks to examine the diagnostic and management strategies presently employed in these clinical settings, while drawing attention to the significant public health implications of KIU, particularly its increasing incidence among younger populations.

## Materials and methods

Study design

This was a multi-center convergent mixed-methods observational study consisting of two components: a cross-sectional knowledge, attitude, and practice (KAP) survey of primary care practitioners and urologists in the Cheshire and Merseyside region and a retrospective review of diagnostic and management pathways for patients with KIU over a six-month period at a high-volume urology center.

Ethical considerations

The protocol for this research project was approved by a suitably constituted local institutional review board and conformed to the provisions of the Declaration of Helsinki (approval no. S367-24-25). Given the retrospective nature of the data collection and the use of anonymized records, informed consent was waived. 

KAP survey

Primary care professionals and urologists practicing in the Cheshire and Merseyside region were invited to complete an online questionnaire. A purposive sampling strategy was employed to target clinicians likely to encounter ketamine uropathy in their practice. 

A structured KAP survey was developed through a comprehensive review of current literature, and survey content was validated by consultation with subject matter experts. The resulting questionnaire was designed to assess three key domains: first, respondents' knowledge of pathophysiology, clinical features, and diagnostic criteria of KIU; second, their awareness of and attitudes toward ketamine misuse and its associated complications; and third, their clinical practice patterns concerning the identification, referral, and management of patients affected by this condition. 

The survey consisted of multiple-choice, Likert-scale, and free-text items and was disseminated using Google Forms between August and September 2024. It was distributed via email to all secondary care professionals working in urology, as well as to all general practices in the Cheshire and Merseyside region. From there, it was further disseminated to general practitioners (GPs), advanced nurse practitioners (ANPs), and primary care nurses working in primary care settings. Survey responses were then analyzed using descriptive statistics. Categorical variables were summarized as frequencies and percentages. Comparative analyses between primary and secondary care responses were conducted using the chi-square test of independence, as appropriate.

Retrospective review

A retrospective review was conducted on all new outpatient referrals and in-hospital admissions for KIU between February and August 2024 within the Mersey and West Lancashire Teaching Hospitals NHS Trust.

Patients were eligible for inclusion if they met the following criteria: individuals of any age with a documented history of ketamine use accompanied by symptoms affecting the upper or lower urinary tract and corroborating evidence from biochemical, radiological, cystoscopic, or histological investigations consistent with KIU. Notably, no exclusion criteria were applied, ensuring that all individuals meeting the inclusion parameters were considered for the study. 

Clinical and diagnostic data were extracted from the local electronic patient record system (Careflow EPR, System C Healthcare Ltd, UK). Operation notes were reviewed using the local electronic theatre system (OPERA software). Data collected included demographic details, laboratory results, imaging reports, histology findings, diagnostic modalities, treatment approaches, referral pathways, and clinical outcomes. Information was abstracted using a standardized data collection form and entered into Microsoft Excel (Microsoft ® Corp, Redmond, WA, USA), stored securely on encrypted institutional servers accessible only to the research team. Identifiable information was anonymized prior to analysis to maintain participant confidentiality. 

Data analysis was performed using IBM SPSS Statistics (Version 30; IBM Corp., Armonk, NY, USA). Descriptive statistics were used to summarize patient characteristics, diagnostic pathways, management strategies, and outcomes.

## Results

KAP survey

A total of 107 healthcare professionals from the Cheshire and Merseyside region participated in the survey. Of these, 28.9% (n=31) were from primary care, including GPs, nurse practitioners, and practice nurses, while 71.1% (n=76) were secondary care professionals working in urology. The majority of secondary care respondents were based at Whiston Hospital, St Helens Hospital, and the Royal Liverpool University Hospital (67.0%), with additional representation from Southport & Ormskirk Hospitals (11.0%), Leighton Hospital (7.0%), Countess of Chester Hospital (6.0%), Arrowe Park Hospital (4.0%), Wrexham Maelor Hospital (3.0%), and Warrington Hospital (2.0%). The distribution of respondents by healthcare sector and professional role is detailed in Table 1.

**Table 1:** Distribution of survey respondents by healthcare sector and role

Healthcare sector	Professional role	Number of respondents (%)
Primary care	Primary care nurse/nurse practitioner	14 (13.0)
	General practitioner	17 (15.9)
Secondary care	Secondary care nurse/nurse practitioner	14 (13.1)
	Clinical nurse specialist	6 (5.6)
	Core surgical trainee	5 (4.6)
	Specialty registrar	23 (21.4)
	Consultant	28 (26.4)
Total		107 (100.0)

A summary of all Chi-square analyses conducted to assess associations between professional background and responses to survey items is presented in Table 2.

**Table 2:** Summary of chi-square analyses examining associations between survey variables and the healthcare sector (primary vs. secondary care) Chi-square tests were performed to assess associations between the healthcare sector (primary vs. secondary care) and responses to survey items. df are listed for each test. Values with p<0.05 were considered statistically significant and are indicated in the final column. df, degrees of freedom; KIU, ketamine-induced uropathy

Survey domain	χ² value	df	p-value	Significant (p<0.05)?
Awareness of ketamine misuse	7.626	1	0.005	Yes
Clinical exposure to ketamine users	8.256	3	0.041	Yes
Perceived affected age group	6.981	5	0.072	No
Awareness of KIU guidelines	2.133	1	0.144	No
Quantification of ketamine use	9.725	5	0.045	Yes
Enquiry about sexual function	1.772	5	0.778	No
Initiating investigations in primary care	5.161	7	0.023	Yes
Initial management prior to referral	0.011	8	0.913	No
Referral urgency	0.397	3	0.941	No
Awareness of reconstructive urology services	10.683	1	0.004	Yes
Follow-up preferences post-treatment	0.070	1	0.791	No

Awareness and Clinical Exposure to Ketamine Misuse

Among secondary care respondents, 93.4% recognized ketamine misuse as a significant regional and national concern. In contrast, limited awareness at the regional level was more frequently reported by primary care respondents (61.5%), indicating a potential gap in community-level recognition. Overall awareness of ketamine misuse was significantly higher in secondary care (p=0.005).

**Figure 1:**
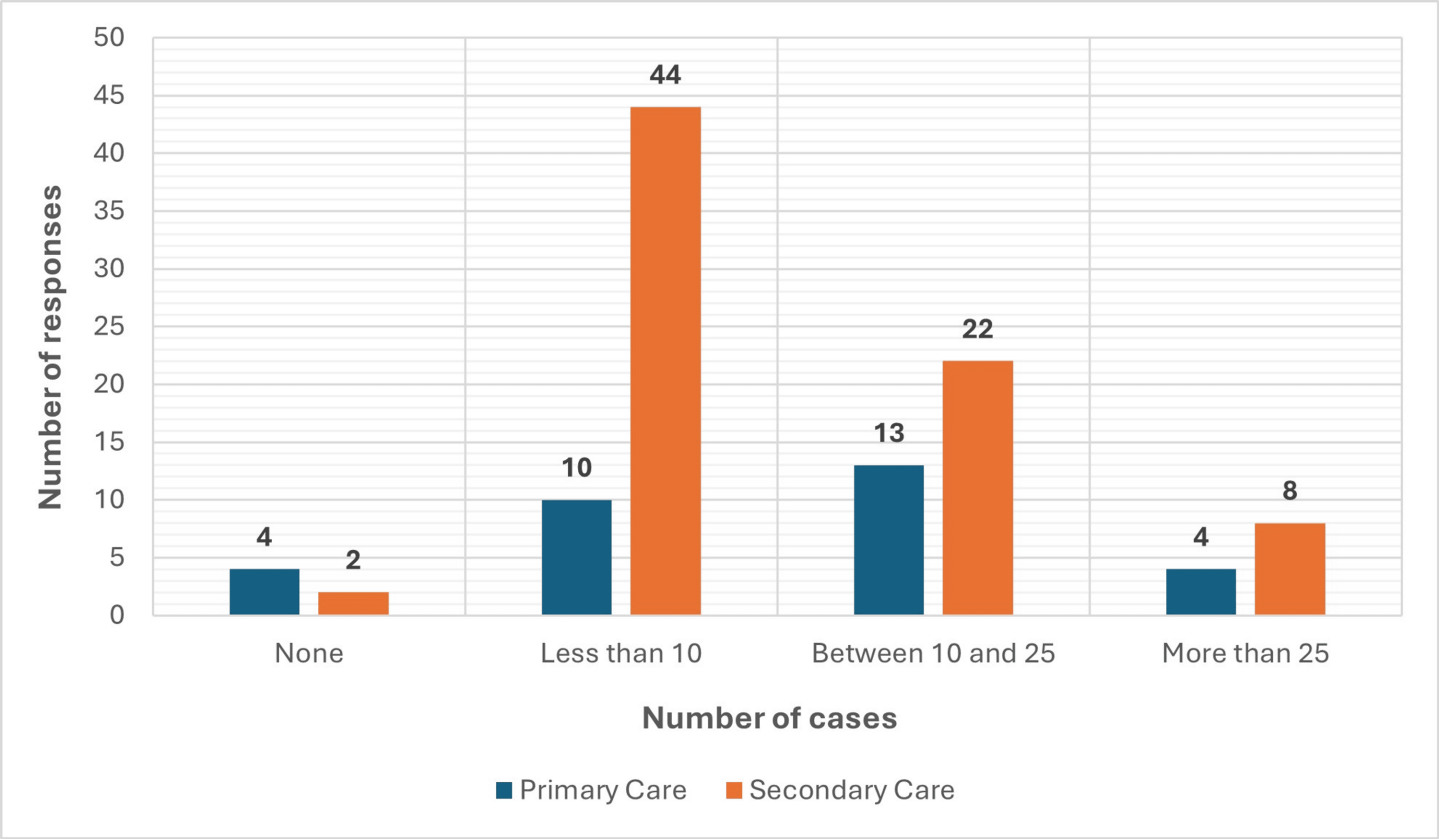
Number of ketamine misuse cases seen in the last year across sectors

As shown in Figure 1, secondary care professionals also reported significantly greater clinical exposure to patients misusing ketamine (p=0.041). GPs and specialty registrars reported the highest encounter rates, with 47.1% and 43.0%, respectively, having seen over 10 such patients in the past year. In contrast, nursing staff and nurse practitioners reported the lowest exposure, highlighting disparities in patient distribution across clinical roles.

Perceived Demographics of Ketamine Misuse

Most respondents (70.1%) identified individuals aged 20-29 as the group most affected by ketamine misuse, followed by 24.3% citing those aged 10-19. This indicates a general consensus that ketamine misuse is primarily a concern among younger populations.

Beliefs About Ketamine Pharmacology and Systemic Effects

Most respondents (93.5%) correctly identified ketamine as an anesthetic, while 68.2% recognized its use as a licensed analgesic, and 66.4% accurately classified it as a Schedule B controlled substance under UK drug legislation. Additionally, 36.5% acknowledged its emerging roles in antidepressant and antiepileptic therapy.

Regarding systemic effects, 86.0% (n=92) identified lower urinary tract involvement, and 84.1% (n=90) recognized central nervous system effects. However, awareness was lower for upper urinary tract (64.5%, n=69) and hepatobiliary complications (49.5%, n=53), indicating knowledge gaps in the broader impact of chronic ketamine misuse.

Awareness of Management Guidelines

Overall, 63.6% of respondents (n=68) reported no awareness of regional or national guidelines for managing KIU. Among those who indicated some familiarity, references were often anecdotal and lacked citations of formal consensus documents. Awareness of the recently published British Association of Urological Surgeons (BAUS) guidelines was low, reported by only 12.9% of primary care respondents and 32.8% in secondary care, primarily among consultant urologists [9]. These findings highlight a substantial gap in guideline dissemination, particularly in community settings.

Clinical Enquiry Into Patterns of Ketamine Use and Related Sequelae

Overall, 50.5% of respondents reported routinely assessing key parameters of ketamine use, such as dosage, frequency, mode of administration, and duration of use, while 27.1% indicated they “rarely” or “never” did so. Of those in the latter group (n=29), 37.9% were from primary care and 62.1% from secondary care, predominantly nurses and junior surgical trainees, suggesting gaps in routine history-taking among certain roles or training levels.

**Figure 2:**
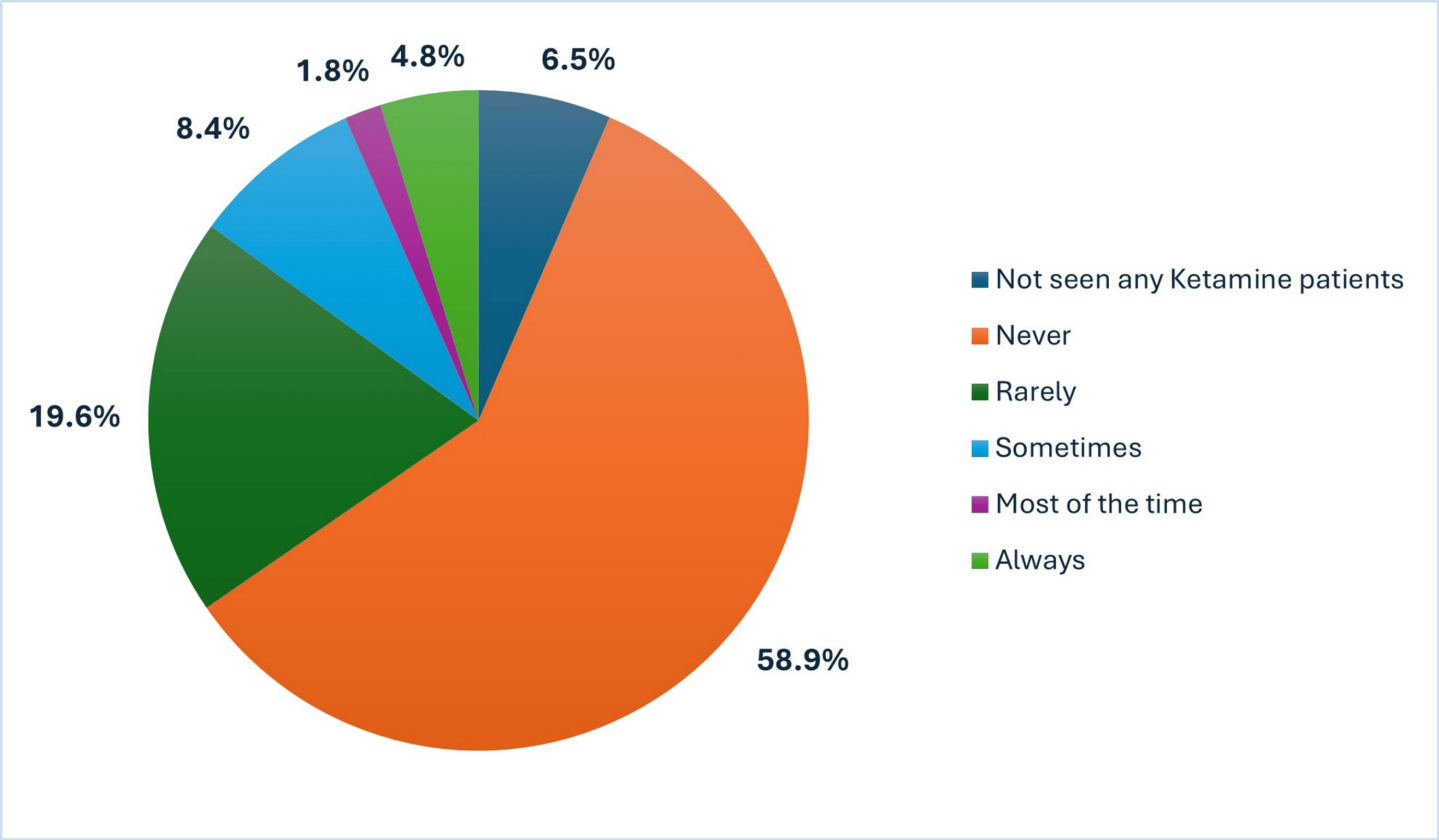
Frequency of clinical enquiry into the impact of ketamine misuse on sexual function

As shown in Figure 2, more than half of respondents (58.9%) reported never enquiring about the impact of ketamine misuse on sexual function, indicating this aspect is often overlooked in clinical assessments.

Secondary care professionals were significantly more likely to quantify ketamine use during evaluations (p=0.045), though no significant difference was found between sectors regarding an enquiry into sexual health impacts (p=0.778).

Investigation and Treatment Patterns in Primary and Secondary Care

**Figure 3:**
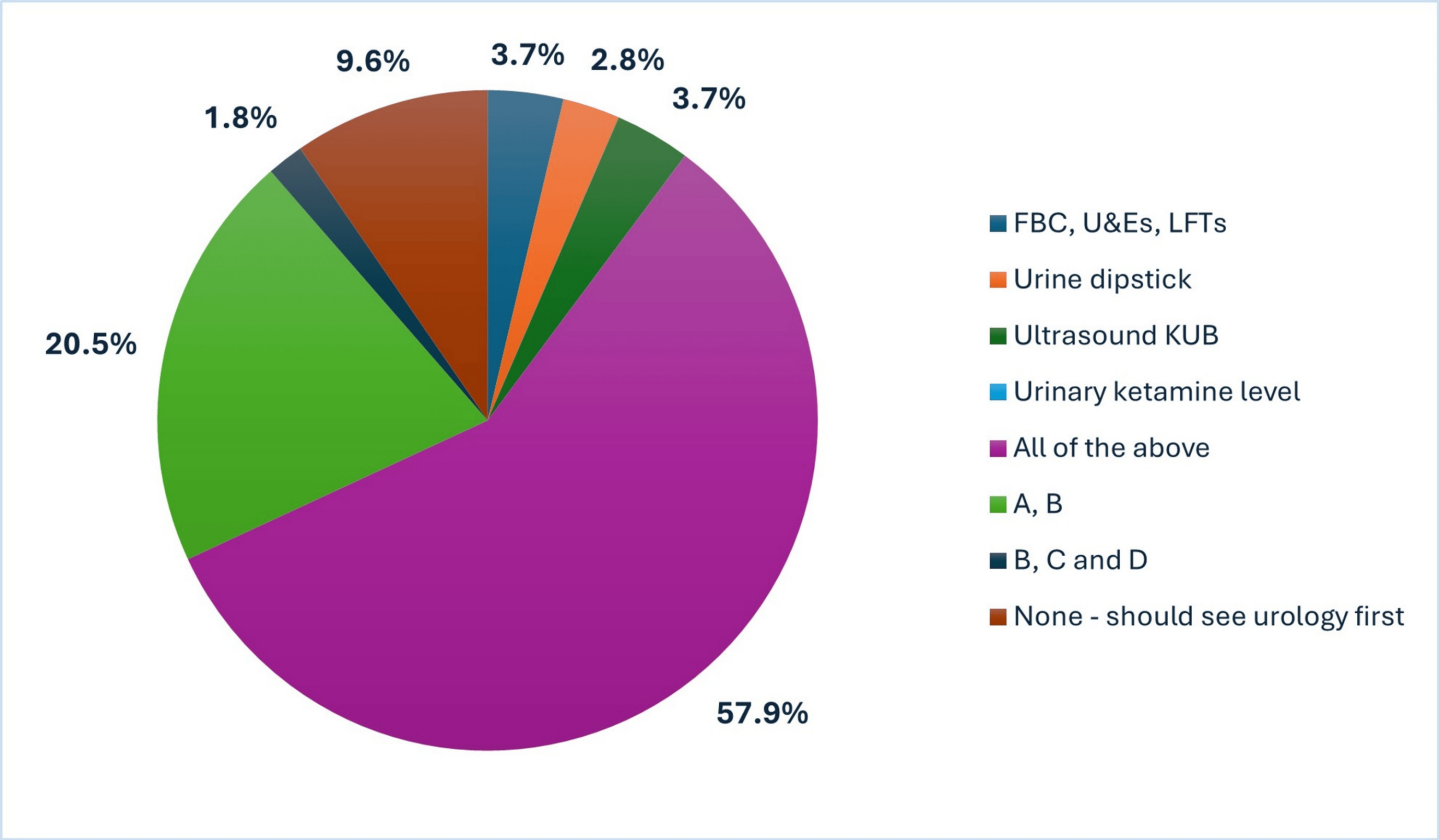
Investigations considered necessary in primary care FBC, full blood count; U&Es, urea and electrolytes; LFTs, liver function tests; KUB, kidneys, ureters, and bladder

**Figure 4:**
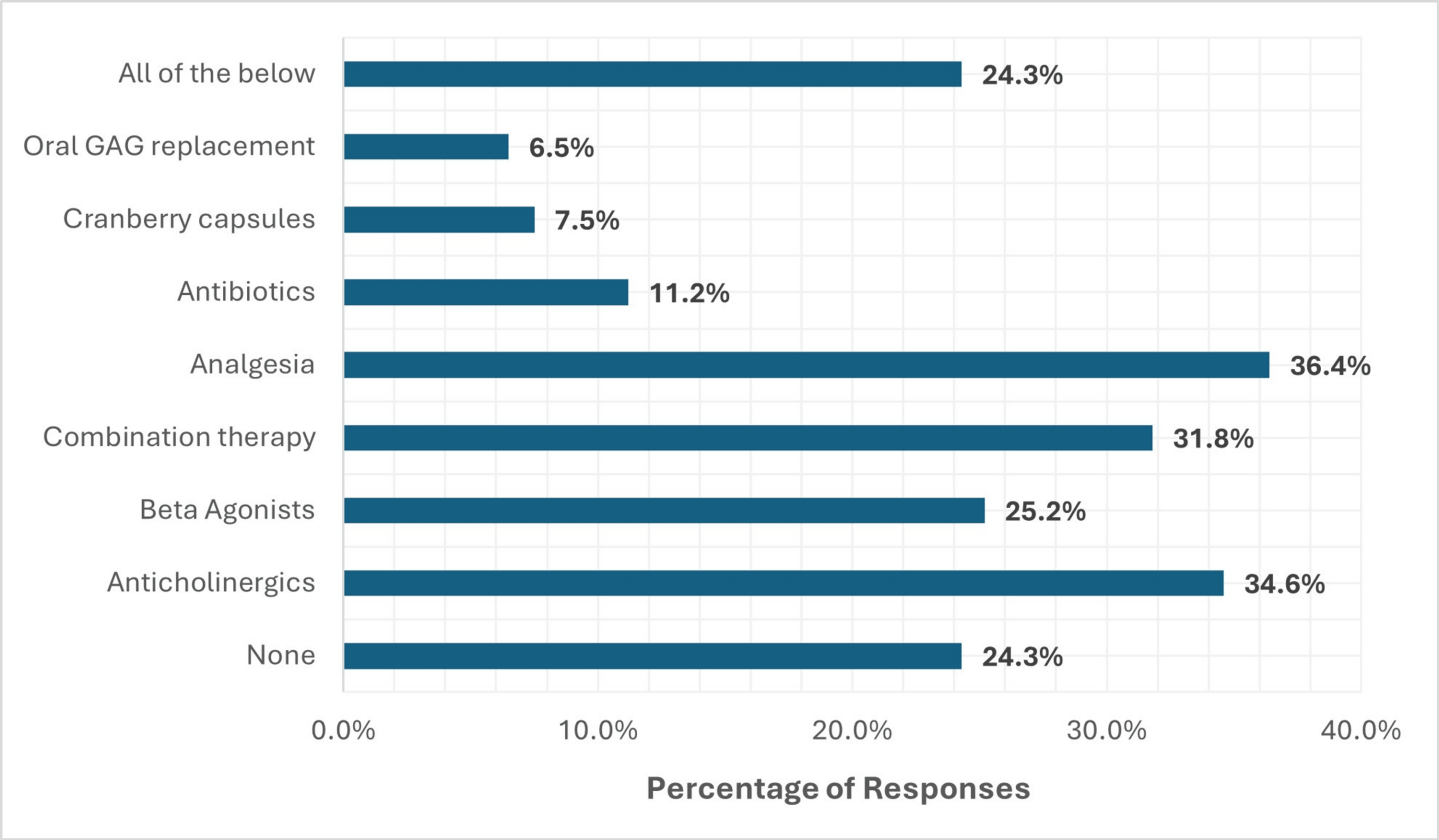
Interventions considered appropriate before urologist referral

Survey responses revealed marked variability in diagnostic and treatment practices for KIU in primary care (Figure 3 and Figure 4). Notably, 9.6% of primary care respondents advocated for a direct referral to urology without prior investigations, while nearly two-thirds supported baseline community workup, including serum and urine analyses, ketamine toxicology, and renal ultrasonography.

In terms of initial management, 24.3% of primary care clinicians expressed a lack of confidence in initiating treatment prior to referral. Concerningly, 11.2% reported prescribing antibiotics for suspected ketamine cystitis, raising concerns about inappropriate antimicrobial use.

In secondary care, 64.5% of respondents routinely arranged renal ultrasonography at first review, with 8.4% preferring advanced imaging such as computed tomography with a urographic protocol or MAG-3 renography. Despite concerns about tolerability among younger patients, 40.2% would proceed with flexible cystoscopy at the initial review. Urodynamic studies were less commonly employed, with 14% opting for standard urodynamics and 2.8% recommending video-urodynamics.

Secondary care respondents were significantly more likely to endorse initiating investigations in primary care (χ²=5.161, p=0.023).

Urgency of Referral Pathways and the Role of Multidisciplinary Care

**Figure 5:**
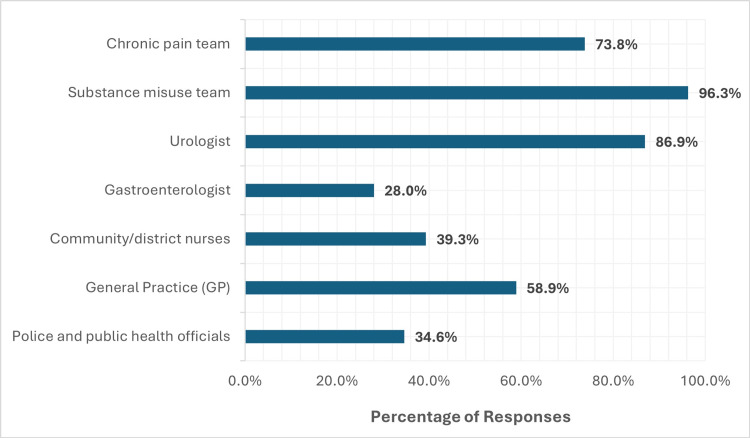
Perceived multidisciplinary teams involved in the management of KIU KIU, ketamine-induced uropathy

A strong consensus emerged in support of a multidisciplinary approach to KIU management, with most respondents endorsing the coordinated involvement of urologists, chronic pain teams, and substance misuse services (Figure 5).

Regarding referral urgency, 58.9%, primarily from primary care, believed patients should be seen by urology within four weeks, highlighting a potential mismatch between perceived urgency and current service capacity. The remaining 41.1% selected longer timeframes (three months to one year).

Most respondents (73.9%) preferred referral to a functional urologist, with 39.3% advocating for a dedicated subspecialty in KIU. Notably, 94.4% supported the development of a dedicated referral pathway to a multidisciplinary clinic incorporating urology, pain management, addiction services, and social support.

Access to Reconstructive Urology Services for KIU

A substantial proportion of respondents (38.3%) were unaware of whether reconstructive urology services for KIU were available at their local hospital. Among those with knowledge of local services, 32.7% reported that no such service existed. Additionally, 28.0% had referred patients to external centers for reconstructive intervention. Awareness of service availability was significantly higher among secondary care professionals (p=0.004).

Post-treatment Follow-Up and Educational Needs in Primary Care

A slight majority (56.1%) supported continued secondary care follow-up for patients with KIU after initial treatment, including nearly all primary care respondents (n=25), reflecting a preference for ongoing specialist involvement. In contrast, 43.9% favored discharge to general practice; however, only six were from primary care, suggesting limited confidence in managing long-term outcomes in the community.

Notably, all 31 primary care respondents expressed interest in attending an educational session led by urology specialists to update community providers on current KIU management strategies.

Retrospective review

A total of 65 patients with KIU were included in this retrospective cohort review. The cohort comprised all new outpatient referrals to urology from GPs or other specialties, as well as all acute hospital admissions recorded over a six-month period (February to August 2024) across two sites within the Mersey and West Lancashire Teaching Hospitals NHS Trust: Whiston Hospital and St Helens Hospital.

The median age of patients was 22 years. Of these, 39 (60.0%) were male and 26 (40.0%) were female. A total of 24 patients (36.9%) had a documented history of behavioral disorders (e.g., autism spectrum disorder and ADHD) or psychiatric conditions (e.g., depression and schizophrenia). Additionally, 30 patients (46.2%) reported concurrent or previous misuse of other substances, including alcohol, cannabis, opiates, cocaine, and benzodiazepines.

The predominant route of ketamine administration was intranasal, reported in 60 patients (92.3%), while oral ingestion was noted in five patients (7.7%). Seventeen patients (26.2%) presented as emergency hospital admissions, of whom four required urgent bilateral nephrostomy insertion due to obstructive uropathy.

The presenting symptoms of KIU varied considerably, and many patients reported more than one symptom concurrently. Table 3 outlines the distribution of these presenting complaints across the study cohort.

**Table 3:** Frequency of reported symptoms among patients diagnosed with KIU KIU, ketamine-induced uropathy

Presenting symptom	Number of patients (%)
Urgency	51 (78.5)
Frequency	43 (66.2)
Dysuria	38 (58.5)
Haematuria	30 (46.2)
Bladder pain	29 (44.6)
Abdominal pain	20 (30.7)
Nocturia	14 (21.5)
Urge incontinence	5 (7.7)
Flank pain	4 (6.2)

Table 4 stratifies the patient cohort according to key parameters related to ketamine misuse, including daily dose (grams), frequency of use, and duration of misuse. At the time of data collection, 13 patients (20.0%) self-reported abstinence from ketamine misuse for at least 30 days. However, it is important to note that no confirmatory toxicology testing was performed to objectively verify abstinence status.

**Table 4:** Key parameters of ketamine misuse among KIU patients *Percentages are based on a total of 65 patients. KIU, ketamine-induced uropathy

Parameter	Unit	Patients, n (%)*
Dose of ketamine/day	≤1 g	9 (13.8)
	2-4 g	38 (58.5)
	≥5 g	18 (27.7)
Frequency of use/week	≤1 day	6 (9.2)
	2-4 days	29 (44.6)
	5-6 days	20 (30.7)
	Daily	10 (15.5)
Duration of misuse	<6 months	5 (7.7)
	6-12 months	16 (24.7)
	12-24 months	24 (36.9)
	>24 months	20 (30.7)

A comparative analysis was conducted using chi-square tests to examine differences in ketamine misuse parameters, including dosage, frequency, and duration of misuse - based on gender (male vs. female) and the presence or absence of comorbid behavioral disorders, psychiatric diagnoses, or a history of other substance misuse. The analysis did not reveal any statistically significant differences across these variables in our cohort (all p-values>0.05).

**Figure 6:**
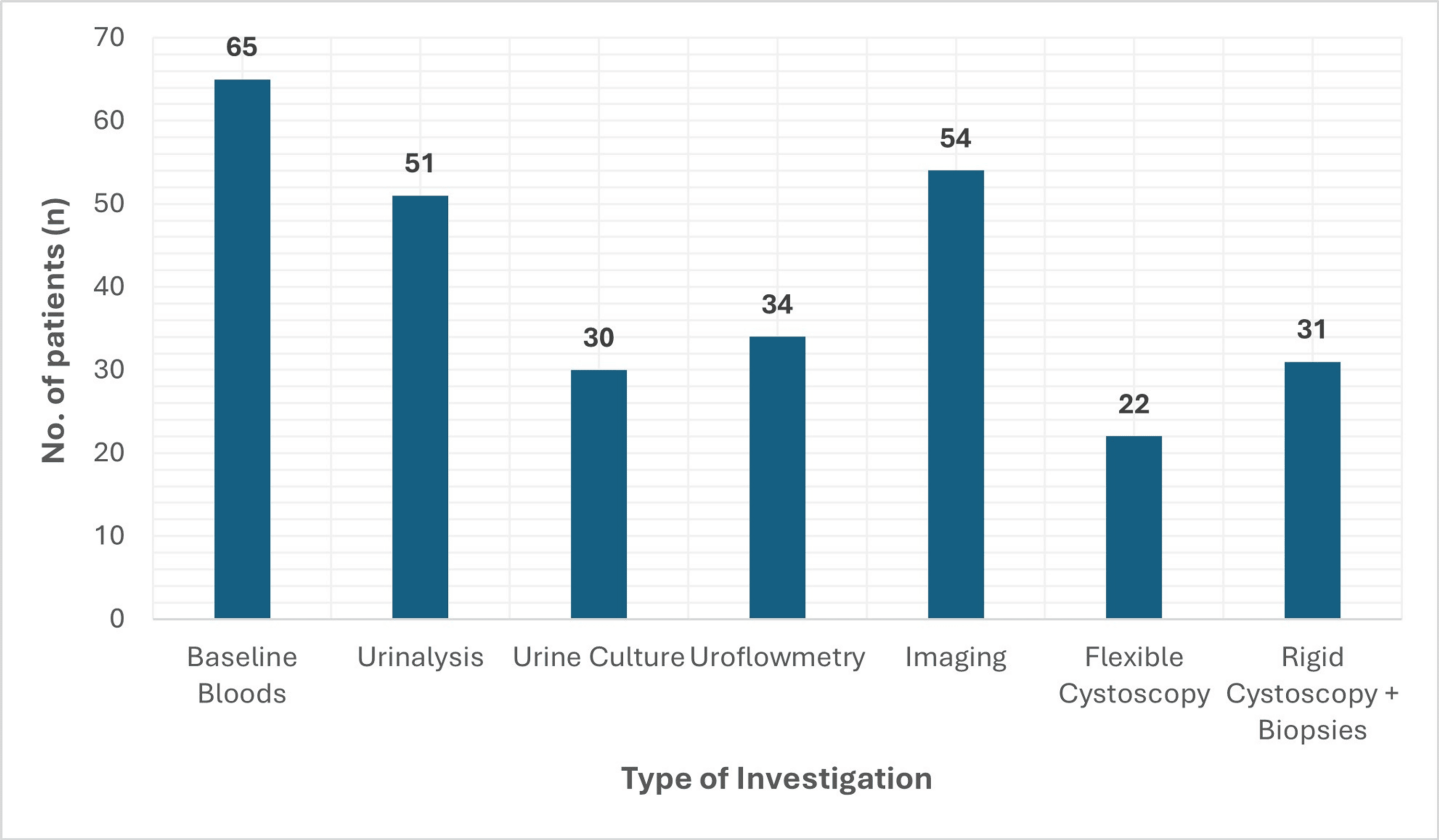
Diagnostic investigations arranged for KIU patients Baseline blood includes full blood count, urea and electrolytes, and liver function tests. Imaging modalities include renal ultrasonography, computed tomography, or MAG-3 renography. KIU, ketamine-induced uropathy

As shown in Figure 6, investigations following initial urology clinic review varied across the cohort. Impaired renal function was observed in nine patients (13.8%), with the lowest eGFR recorded at 26 mL/min/1.73 m². Abnormal liver function tests were present in 27 patients (41.5%), all demonstrating a cholestatic pattern.

Imaging included renal ultrasonography (n=39, 60.0%), computed tomography (n=10, 15.4%), and MAG-3 renography (n=5, 7.7%). Findings included bilateral hydronephrosis in nine patients (13.8%), bladder wall thickening in 17 (26.2%), and reduced bladder capacity in 12 (18.5%).

Flexible cystoscopy was not tolerated in 12 patients. Bladder biopsies were performed in 31 patients (47.7%), all showing histological features of chronic inflammation consistent with ketamine-induced cystitis.

**Figure 7:**
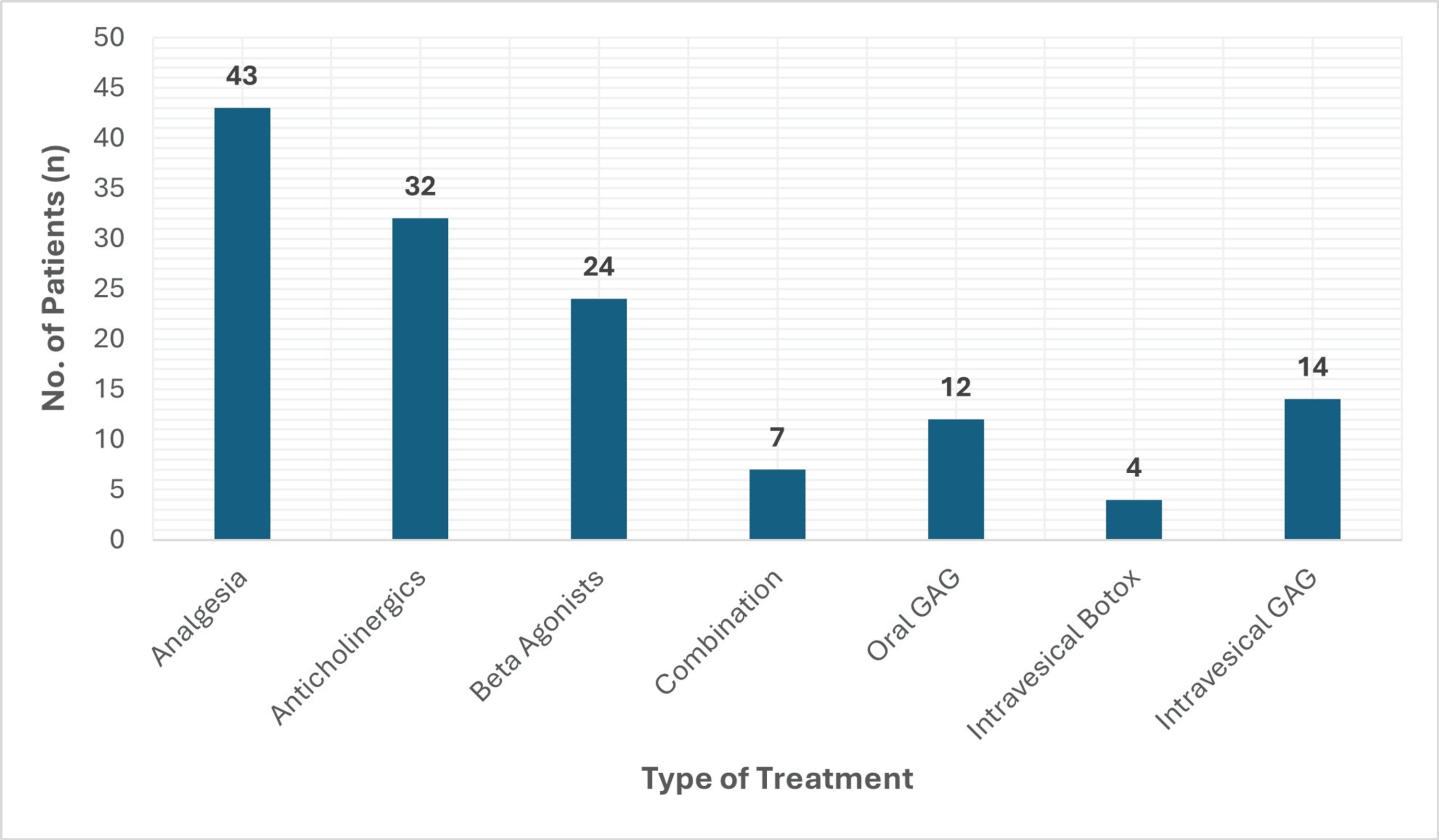
Treatment regimens offered to KIU patients KIU, ketamine-induced uropathy

Considerable variability was observed in the treatment regimens offered to patients, as summarized in Figure 7. Analgesic options included paracetamol, non-steroidal anti-inflammatory drugs (NSAIDs), weak opioids (e.g., codeine), and gabapentinoids. Anticholinergic therapy primarily involved either Solifenacin or Oxybutynin, while β₃-adrenoceptor agonist therapy consisted of Mirabegron. Intravesical treatments were reserved for patients who had undergone cystoscopic evaluation, bladder biopsy, and had histologically confirmed ketamine-induced cystitis.

A total of 54 patients (83.1%) were referred to either drug and alcohol services or children’s safeguarding, based on age. Forty-seven patients (72.3%) were referred to the chronic pain team for analgesic support, while 20 patients (30.7%) required gastroenterology input due to abnormal liver function tests. Fourteen patients (21.5%) were referred to dietetic services for low body mass index (BMI ≤18.5).

Non-attendance at outpatient appointments was common: 27 patients (41.5%) missed at least one appointment related to their KIU management, and 13 patients (20.0%) missed two. Five patients (7.7%) were discharged back to primary care due to repeated non-attendance.

## Discussion

Ketamine misuse is a rapidly escalating public health concern in the United Kingdom. According to data from the UK Office for National Statistics, ketamine use has increased by 148.7% since 2013, with a disproportionate burden in the northwest of England, particularly in Liverpool and Manchester, recording the highest number of drug-related offenses nationally (12.1 and 8.2 per 1,000 people, respectively) [7]. This dramatic rise has overwhelmed frontline healthcare services and underscored the urgent need to evaluate how well-prepared clinicians are in identifying, managing, and preventing KIU. The present study was conducted in response to this regional crisis, aiming to explore the KAP of healthcare professionals across Cheshire and Merseyside in managing KIU and its associated complications.

Awareness disparities and the role of primary care

Our findings reveal a critical under-recognition of the role of primary care in managing KIU. Only 58.9% of survey respondents believed that GPs should be involved in the care of these patients, reflecting an alarming gap in awareness regarding the importance of early detection and primary prevention. This is consistent with prior literature that emphasizes the pivotal role of community-based interventions in substance misuse disorders [10,11]. Early-stage identification and management are known to reduce progression to irreversible uropathy, thereby limiting the need for interventions such as nephrostomy, urinary diversion, or cystectomy [12,13].

Confidence in arranging investigations or initiating treatment was significantly lower in primary care respondents, in contrast to secondary care professionals, particularly consultants and specialty registrars, who demonstrated greater clinical exposure and familiarity with guideline-based management. Specialty registrars, in particular, reported high encounter rates due to their front-line roles in acute urology, consistent with literature noting that junior clinicians often function as first-line triage in emergency settings [14,15].

Knowledge gaps and the need for structured education

The limited awareness of formal guidelines, including the BAUS 2024 consensus on KIU, was a key finding. Only 12.9% of primary care and 32.8% of secondary care respondents reported familiarity with these guidelines, highlighting opportunities to enhance the dissemination and uptake of evolving clinical standards. Similar disparities have been noted in the uptake of guidelines in other substance-related disorders [16,17].

Educational sessions led by urologists and targeted at primary care teams could address this by enhancing awareness of KIU-specific investigations, management strategies, and appropriate referral pathways. An improved understanding of ketamine’s systemic effects, particularly hepatobiliary complications, could also facilitate the timely involvement of gastroenterology teams, a recommendation supported by emerging evidence linking ketamine to cholangiopathy and liver dysfunction [18,19].

Enhanced training should also emphasize the importance of thorough clinical history-taking. Parameters such as dosage, route, frequency, and duration of ketamine use, as well as associated complications like sexual dysfunction, are essential in staging disease severity and determining the appropriate diagnostic and therapeutic approach [20].

Diagnostic and therapeutic challenges

A higher proportion of secondary care respondents reported using investigations aligned with the new BAUS guidance, such as renal imaging and cystoscopy. However, flexible cystoscopy and uroflowmetry were frequently not tolerated by young patients, a trend also reported in recent clinical reviews [12]. In such cases, clinicians may reasonably consider rigid cystoscopy under general anesthesia, emphasizing the importance of individualized clinical judgment [9].

Treatment variability was observed across both sectors, although secondary care providers more consistently employed first-line therapies such as anticholinergics (e.g., Solifenacin), β₃-adrenoceptor agonists (e.g., Mirabegron), and analgesics. In contrast, 11.2% of primary care clinicians reported prescribing antibiotics for suspected ketamine cystitis, raising concerns about inappropriate antimicrobial use, consistent with broader trends in antimicrobial stewardship gaps [21].

Gaps in service awareness and regional provision

Awareness of reconstructive urology services was limited, with 38.3% of all respondents unsure whether such services existed at their institutions. Among those who were informed, 32.7% reported no access to reconstructive options locally, underscoring major gaps in regional service provision. This highlights the urgent need for regional mapping and development of formal referral pathways, in line with recommendations for equitable access to specialist care [22].

Multidisciplinary care and referral urgency

Support for multidisciplinary management was nearly unanimous among respondents, with most advocating for integrated care involving urologists, chronic pain specialists, addiction services, and psychosocial support. However, a mismatch was evident between the perceived urgency of urology referrals (with 58.9% believing patients should be seen within four weeks) and the current service delivery capacity. Multidisciplinary clinics dedicated to KIU, with ring-fenced resources and defined care pathways, may be a viable solution to address this gap and provide holistic, biopsychosocial care [23].

Additionally, a notable proportion of respondents believed that police and public authorities should be involved in the broader management of ketamine misuse. This reflects the wider societal burden posed by ketamine abuse, particularly its disproportionate impact on younger populations and its intersection with issues of criminal justice and public health [7].

Non-attendance and psychosocial considerations

Non-attendance was a recurring issue in the retrospective cohort, with over 40% of patients missing at least one follow-up appointment. This poses a challenge not only to timely care but also to the efficient use of healthcare resources. Early involvement of addiction services and safeguarding teams may mitigate these barriers by establishing a supportive framework to improve patient adherence, a strategy supported by evidence in other substance use contexts [24,25].

Continuity of care and long-term management

While 56.1% of respondents supported ongoing follow-up in secondary care, others favored eventual discharge to general practice. The decision to continue specialist oversight should be individualized, informed by the severity of symptoms, investigation results, and overall social context. Ultimately, the goal remains to protect renal and hepatic function, facilitate cessation of ketamine use, and restore psychosocial well-being.

Future directions: a case for subspecialization

Given the rising incidence and complexity of KIU, there is a compelling rationale for the development of a dedicated subspecialty within functional urology. Subspecialization would enable centralized clinical expertise, coordinated multidisciplinary care, and a structured research agenda, mirroring the evolution seen in other complex functional disorders [26].

Limitations

While the survey captured a diverse cohort of regional healthcare professionals, potential self-selection bias may have influenced response patterns, with those more engaged or aware of KIU perhaps more likely to participate. This is also reflected in the disproportionate representation of secondary care respondents relative to primary care, potentially due to logistical challenges in accessing some primary care practices. Furthermore, self-reported practices may differ from actual clinical behavior, raising the possibility of reporting bias, as suggested in a previous KAP study [27]. The retrospective cohort data, though clinically informative, lacked toxicological confirmation of abstinence and relied on patient self-reporting, which may under- or overestimate true usage patterns. Finally, as the retrospective cohort study covered only a six-month period, this may limit the representativeness and generalizability of the findings and could overlook seasonal trends in drug use. 

## Conclusions

This study underscores the urgent need for system-wide improvements in managing KIU, particularly in high-prevalence regions of the UK. Structured educational initiatives, improved service awareness, and multidisciplinary collaboration are essential to ensure early detection, standardized management, and long-term support. Regional efforts are already underway to raise awareness, not only amongst primary and secondary healthcare professionals but also within local schools and universities, through coordinated public health campaigns and active involvement from the local authorities. As ketamine misuse continues to rise, KIU demands recognition as a serious and complex condition requiring coordinated action across all levels of care.

## Appendices

KAP survey items

1. If in primary care - what is your referring hospital?

- Arrowe Park Hospital

- Wrexham Maelor Hospital

- Countess of Chester Hospital

- Royal Liverpool University Hospital

- Whiston/St Helens Hospitals

- Southport/Ormskirk Hospitals

- Warrington Hospital

- Leighton Hospital

- I do not work in primary care

2. If in secondary care - which hospital are you currently based at?

- Arrowe Park Hospital

- Wrexham Maelor Hospital

- Countess of Chester Hospital

- Royal Liverpool University Hospital

- Whiston/St Helens Hospitals

- Southport/Ormskirk Hospitals

- Warrington Hospital

- Leighton Hospital

- I do not work in secondary care

3. What is your current role?

- General Practitioner (GP)

- Advanced Nurse Practitioner (ANP)/Nurse Practitioner (NP)

- Consultant

- Specialty Registrar

- Clinical Nurse Specialist (CNS)

- Core Surgical Trainee (CST)

- Nursing Staff

4. Are you aware of ketamine misuse in our region/across the country?

- Yes

- No

5. How many patients with ketamine misuse have you seen in the last year, if any?

- None

- Less than 10

- Between 10 and 25

- More than 25

6. Which of these do you think is true regarding ketamine? (tick all that apply)

- Ana=esthetic drug

- Licensed painkiller

- Class B drug

- Anti-depressant

- Anti-epileptic

7. Which body system do you think ketamine affects? (tick all that apply)

- Lower urinary tract system

- Upper urinary tract system

- Hepatobiliary system

- Central nervous system

8. Which age group do you think is most affected by ketamine at present?

- Age 10-19

- Age 20-29

- Age 30-39

- Age 40-49

- Age 50-59

- Age 60 and above

9. Are you aware of any management guidelines for ketamine-induced uropathy?

- Yes

- No

10. If you answered yes to the above question, please specify: (free-text)

11. What symptoms/signs do you think ketamine uropathy can cause? (tick all that apply)

- Lower urinary tract symptoms (urgency, frequency, dysuria, etc.)

- Hematuria

- Bladder/flank pain

- Urinary retention

- Renal failure

12. When seeing suspected ketamine uropathy patients - how often do you ask about dose, frequency, mode of administration, and duration of ketamine use/abstinence?

- Always

- Most of the time

- Sometimes

- Rarely

- Never

- I have not seen any ketamine patients yet

13. Have you ever asked about the impact ketamine misuse can have on sexual function?

- Always

- Most of the time

- Sometimes

- Rarely

- Never

- I have not seen any ketamine patients yet

14. Which specialty do you think should manage ketamine misuse patients? (tick all that apply)

- Chronic pain team

- Substance misuse team

- Urologist

- Gastroenterologist

- Community nurses/district nurses

- General Practitioner (GP)

- Police and public health officials

15. How urgently do you think ketamine uropathy patients should see a urologist?

- Within 4 weeks

- Within 3 months

- Within 6 months

- Within a year

16. Which treatment do you think primary care can prescribe to help ketamine uropathy patients suffering with urgency, frequency, and urinary incontinence before they get to see a urologist? (tick all that apply)

- None, they should see a urologist first

- Anticholinergic agents (e.g. Solifenacin, Darifenacin)

- Beta agonist agents (e.g. Mirabegron)

- Combined therapy (anticholinergic + beta-agonist)

- Pain killers

- Antibiotics

- Cranberry capsules

- Oral GAG therapy

- Can try all of the above (B-G)

17. What investigations, if any, do you think primary care should order for ketamine uropathy patients before they get to see a urologist?

- FBC, U&E, LFTs

- Urine dipstick

- Ultrasound KUB

- Ketamine level in urine testing

- All of the above

- A and B

- B, C, and D

- None - should see a urologist first

18. For secondary care - which of the following would you consider arranging and/or booking in your first clinic review for patients with suspected ketamine uropathy? (tick all that apply)

- IPSS scoring

- ICIQ-LUTS scoring

- 3-day bladder diary

- FBC, U&Es, LFTs

- Urine dipstick

- Ultrasound KUB

- Ketamine level in urine testing

- Urodynamics

- Video-assisted urodynamics

- Flexible cystoscopy

- CT urogram / MAG3 renogram

- All of the above

- None of the above

19. Do you think the management of ketamine uropathy overall should be offered by:

- Any urologist

- Those with an interest in functional urology

- New subspecialist interest in ketamine uropathy

20. Do you agree that ketamine uropathy patients should be seen by a dedicated urologist with subspecialist interest in a joint clinic with multiple disciplines (nurse, pain and substance misuse teams, etc.)?

- Yes

- No

21. Do you have a urologist at your local hospital who offers definitive reconstructive surgery for ketamine uropathy patients?

- Yes

- No

- I do not know

22. Have you ever referred a ketamine uropathy patient to another unit for reconstructive surgery?

- Yes

- No

23. Do you think such patients should be discharged to GP when treated or stay under secondary care?

- Can be discharged to GP when treated

- Should remain under secondary care

24. For primary care - would you welcome an educational event/evening by urology regarding ketamine uropathy management updates for general practice and community nursing?

- Yes

- No
